# Task and Spatial Frequency Modulations of Object Processing: An EEG Study

**DOI:** 10.1371/journal.pone.0070293

**Published:** 2013-07-31

**Authors:** Matt Craddock, Jasna Martinovic, Matthias M. Müller

**Affiliations:** 1 Institute of Psychology, University of Leipzig, Germany; 2 School of Psychology, University of Aberdeen, United Kingdom; Cuban Neuroscience Center, Cuba

## Abstract

Visual object processing may follow a coarse-to-fine sequence imposed by fast processing of low spatial frequencies (LSF) and slow processing of high spatial frequencies (HSF). Objects can be categorized at varying levels of specificity: the superordinate (e.g. animal), the basic (e.g. dog), or the subordinate (e.g. Border Collie). We tested whether superordinate and more specific categorization depend on different spatial frequency ranges, and whether any such dependencies might be revealed by or influence signals recorded using EEG. We used event-related potentials (ERPs) and time-frequency (TF) analysis to examine the time course of object processing while participants performed either a grammatical gender-classification task (which generally forces basic-level categorization) or a living/non-living judgement (superordinate categorization) on everyday, real-life objects. Objects were filtered to contain only HSF or LSF. We found a greater positivity and greater negativity for HSF than for LSF pictures in the P1 and N1 respectively, but no effects of task on either component. A later, fronto-central negativity (N350) was more negative in the gender-classification task than the superordinate categorization task, which may indicate that this component relates to semantic or syntactic processing. We found no significant effects of task or spatial frequency on evoked or total gamma band responses. Our results demonstrate early differences in processing of HSF and LSF content that were not modulated by categorization task, with later responses reflecting such higher-level cognitive factors.

## Introduction

A central goal of vision is to recognize objects rapidly and efficiently. Object recognition may be accomplished through a rapid, automatic cascade of feedforward [1] and feedback [2] operations that culminate in the matching of a processed retinal image to an object representation stored in memory [3]. One general organizing principle of the categorization process may be that it follows a coarse-to-fine sequence imposed by processing of different spatial frequencies at different rates in different visual pathways [4,5]. Many aspects of visual categorization have been shown to follow a general coarse-to-fine, low-to-high spatial frequency sequence [6–8]. In a prominent model of the differing roles of low (LSFs) and high spatial frequencies (HSFs) in object recognition proposed by Bar [2,9,10], LSFs are rapidly projected to frontal cortices via the magnocellular pathway. A guess as to the identity of an object is made via this pathway, and guides subsequent processing of HSFs in inferotemporal cortices through a feedback loop. Thus, LSFs constrain the range of object representations which must be examined in order to find an appropriate match. How might LSFs play such a role in this processing sequence?

One possibility is that LSFs convey the global shape of an object, which guides subsequent “filling-in” by HSF processing. Although both LSFs and HSFs can convey the general shape or spatial layout of an object or scene, LSFs may provide a more stable, less noisy impression of general shape than HSFs [11], and may support the *global precedence* effect seen with hierarchical forms. Typically, participants are able to recognise the global form faster than the local forms [12]; removal of LSF attenuates this advantage [13,14], suggesting that LSF are processed rapidly and aid apprehension of the global form. However, the categorization of global and local forms in compound stimuli is quite different from the categorization of more natural stimuli such as individual objects or scenes [15]. In the case of individual everyday objects, one is rarely challenged to identify either the whole object or the parts that constitute it.

Visual categorization of more natural stimuli often takes place at a hierarchy of levels of specificity. The fastest categorizations are typically made at an intermediate level of specificity called the *basic* level (e.g. ‘dog’) [16], while categorization at more general, *superordinate* levels (e.g. ‘animal’) or more specific, *subordinate* levels (e.g. ‘Shih-Tzu’) [16–19] is often slower [20] (however, see also 21), requiring additional semantic or perceptual processing [17]. Nevertheless, during superordinate categorization of scenes, differences between target and non-target trials have been observed in event-related potentials (ERPs) 150 ms after stimulus onset [22,23]. Furthermore, Macé et al. [24] found that participants made superordinate category judgements for objects presented in scenes faster than basic level category judgements of the same objects. One possibility is that a coarse perceptual representation sufficient to make a superordinate categorization is available rapidly, with further lexico-semantic processing required to make a verbal response. Thus, typical observations of slower superordinate than basic-level category naming may reflect later semantic processes associated with the retrieval of names rather than early perceptual categorizations.

Both accounts assume a fixed temporal order in which categorization first takes place at an intermediate or general level before categorization at other levels, to some extent paralleling the coarse-to-fine processing account. Thus, a potential role of LSF may be to provide an early, coarse representation based on global shape that would support categorization at the first level. Removing LSF may therefore impair categorization at that level, since it slows apprehension of global shape. Furthermore, if subsequent categorizations require prior categorizations made at the first level, the same effects of spatial frequency observed at that level should propagate to each subsequent level.

An alternative to the coarse-to-fine processing account suggests that the order of processing of spatial frequencies is not fixed but moderated by task demands [25–28]. The most useful information for different tasks may be contained in different ranges of spatial frequencies. With faces, the expression, identity, and gender of a face are associated with information at different spatial scales, and attending to those different aspects of faces biases attention to those spatial frequency ranges [26]. Additionally, with hierarchical forms, Shulman and Wilson [29] found that guiding attention to global or local levels facilitated processing of LSF or HSF, respectively. Thus, if different spatial frequency ranges are more informative for categorization at different levels, then asking participants to categorize objects at different levels may influence the order in which different spatial frequencies are processed.

Behavioural evidence on how spatial frequency and level of categorization interact is both limited and mixed. Collin and McMullen [30] presented HSF only, LSF only, and unfiltered objects to participants for categorization at all three levels of specificity. Both subordinate and basic-level categorizations were impaired with LSF-only images, suggesting that categorization at one level may be contingent upon categorization at the other, and that categorization at both levels benefited more from the presence of HSF than LSF information. However, there were no effects of spatial filtering on superordinate categorization. Thus, superordinate categorization is not contingent on prior categorization at a different level, and did not depend more on one range of spatial frequencies than another. On the other hand, while Harel and Bentin [31] also found that subordinate categorization was impaired by the removal of HSF, they found that basic level categorization was equally impaired by removal of either HSF or LSF. Thus, in their study, the combination of HSF and LSF was required to facilitate basic-level categorization. However, they did not examine superordinate categorization performance.

Taken together, the behavioural data from these studies suggest flexibility in the categorization process that is perhaps overlooked by considering it through the lens of a fixed coarse-to-fine order of processing of spatial frequencies, or a fixed temporal order from one level of categorization to another. However, they clearly highlight that different spatial frequency ranges may be useful at different levels of categorization, and thus that the demands of the categorization task may therefore attract attention or processing to different spatial frequency ranges.

Event-related potentials (ERPs) and time-frequency analysis provide more fine-grained temporal detail regarding when and where in visual processing differences may arise. ERP studies have previously revealed both effects of categorization task on early and late visual processing [32,33], and interactions between task and spatial frequency for faces [34]. Tanaka et al. [33] found a greater early negativity (N1; approx. 150 ms) at left posterior sites during subordinate categorization than during basic or superordinate categorization, suggesting that greater analysis of fine visual detail was required for subordinate categorization. A later (approximately 300-350 ms) frontal negativity arose for superordinate relative to basic categorization, which indicated greater involvement of semantic processing for superordinate categorization [35].

Martinovic, Gruber, and Müller [32] found that a covert naming task in which participants reported the grammatical gender of objects also elicited a greater negativity at the N1 compared to superordinate categorization. This early difference is consistent with greater perceptual processing for more specific levels of categorization. They also examined activity in the evoked gamma band (eGBA; 50-150 ms, 30-40 Hz), and found no task-related differences. The lack of a task modulation of eGBA was in keeping with previous results showing its moderation by low-level factors such as image complexity rather than higher-level conceptual processing [36–38]. However, Martinovic et al. did not assess differences in later frontal components of the ERP or in the induced gamma band (iGBA), a later (approximately 200-300 ms), transient, high-frequency (30-90 Hz) response thought to be associated with the activation of high-level object representations.

Spatial frequency was not manipulated in these experiments, but, as argued above, may be an influential factor in categorization at different levels. Goffaux et al. [34] found that the amplitude of the N170 response to faces was enhanced for LSF faces when categorizing them by gender, but did not differ between HSF and LSF faces when rating the familiarity of the faces. Thus, the resources devoted to processing a specific range of spatial frequencies appeared to be moderated by how informative they were for the task. However, it is not clear whether such effects would occur during categorization of non-face objects, given that faces may use specialized processing mechanisms [34,39,40].

We used ERPs and time-frequency analysis in the human electroencephalogram to examine the time course of object categorization at different spatial frequencies during different categorization tasks. Specifically, we contrasted responses to familiar objects containing different spatial frequencies – specifically, HSF and LSF pictures – during a grammatical gender-classification task and a living/non-living superordinate categorization task. Our stimuli were filtered to contain frequency ranges that have been previously shown to be preferentially processed by different visual pathways [9]. Fintzi and Mahon [41] have shown that the orbito-frontal cortex/magnocellular route proposed as critical by Bar et al [9]. responds most strongly to stimuli containing spatial frequencies between 0.17 and 0.38 degrees of visual angle, while ventral areas such as LOC showed stronger preferences for stimuli containing spatial frequencies above 4.76 cycles per degree of visual angle. The precise ranges used in other experiments [9,30,34] which manipulate spatial frequency in categorization are variable, but in general exclude frequencies above approximately 1.2 cycles per degree (cpd) of visual angle for LSF pictures and below approximately 4 cpd for HSF images.

We used the same grammatical gender classification task used previously in EEG studies of visual object recognition [32,38], in which participants must generally categorize objects at the basic level to successfully decide the grammatical gender of the object’s name. The additional load of making the grammatical decision should not influence early processes, which should focus on making an initial categorization. Such tasks avoid problems related to production of vocal responses during recording of EEG, and, as noted above, have previously been shown to elicit ERP differences in the visual N1. We restricted the stimuli to objects with masculine or feminine gendered names so that both tasks had only two possible responses.

In the time-frequency domain, we concentrated on activity in the gamma band range. As noted above, both eGBA and iGBA have been associated with object recognition. eGBA is highly sensitive to low-level stimulus properties such as complexity [38] and size [42], whereas iGBA is typically unaffected by such properties. Both are included in measures of *total* GBA (tGBA). Although previous research related induced gamma band to the activation of an object representation, it was often confounded with an artefact of miniature eye movements [43]. Gamma band activity seems to retain its sensitivity to objects [44,45] after correction, which we apply here. However, its role is as yet unclear [46]. We hoped that analysis of changes in gamma band power may further elucidate its role in object recognition. If it reflects primarily high-level object representation, as previously thought, then it should be relatively unaffected by perceptual manipulations such as spatial frequency, but may be modulated by task. Thus, manipulating both task and perceptual factors may allow us to differentiate between high-level and low-level roles for gamma band activity in object categorization.

We expected that if different spatial frequency information were preferentially processed and used for categorizations at different levels of specificity, we would see early interactions between task and spatial frequency. Alternatively, if the initial categorization is always made at the same level of specificity regardless of task, or if both superordinate and gender classification require information from the same range of spatial frequencies, then we would not expect such an interaction. If early ERPs largely reflect perceptual processing, then we may also see early effects of spatial frequency during both tasks. We also expected a later, frontal negativity during superordinate categorization, as observed by Tanaka et al. [33]. If this negativity is primarily semantic and arises when perceptual analysis is relatively complete, then it should not be influenced by spatial filtering. Furthermore, we also expected to see effects of task in another late ERP component, the late positive complex; this component has been related to secondary categorization processes such as name retrieval [47], and may therefore differ across tasks.

## Methods

### Participants

Sixteen participants (age range 18 to 41 years, mean = 26 years) were recruited from the participant database of the University of Leipzig EEG Laboratory and received a course credit or a small honorarium for participating. All participants reported normal or corrected-to-normal vision. Individual written informed consent was obtained. The study conformed to the Code of Ethics of the World Medical Association and was approved by the local ethics committee of the University of Leipzig.

### Stimuli and apparatus

Stimuli were 296 greyscale photographs taken from a commercial image database (Hemera Photo Objects). Many were used in a previous study [32]. Half of these stimuli depicted living objects and half depicted non-living objects. Each 400 × 400 pixel image showed a single object in isolation against a neutral grey background. The stimuli were presented on a 17 inch monitor with a screen resolution of 1024 × 768 pixels (refresh rate 85 Hz; note that due to a technical issue, the refresh rate was 75Hz for several participants). Participants sat approximately 80 cm in front of the monitor in a dimly lit testing chamber. The stimuli (the object and background) thus subtended approximately 10 degrees of visual angle in each direction. The order and timing of the presentation of images was controlled using the Cogent toolbox for Matlab (Cogent, www.vislab.ucl.ac.uk/Cogent/; The Mathworks, Inc, Natick, Massachusetts).

**Figure 1:**
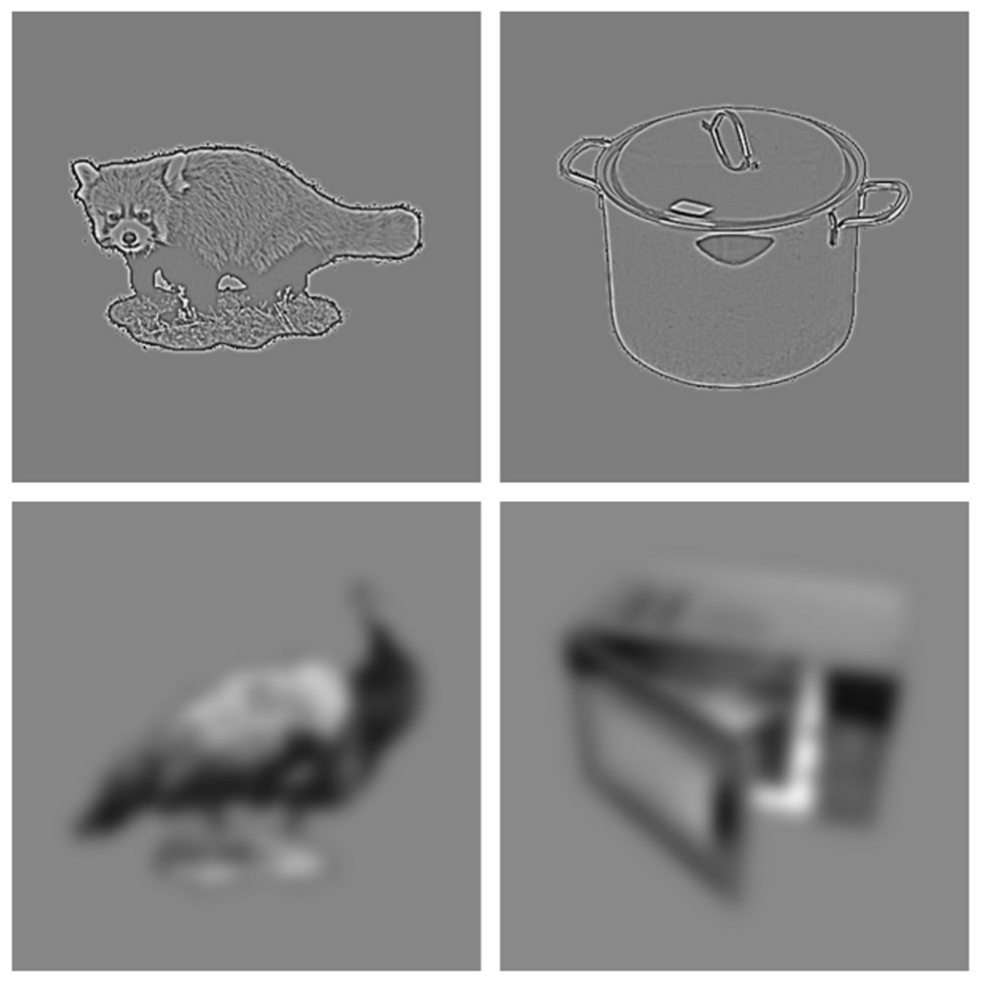
Example stimuli after filtering. Examples of high-pass (HSF) and low-pass (LSF) filtered images of living and non-living objects.

High and low spatial frequency filtered images were produced by multiplying the fast Fourier transformed amplitude spectrum of each image with a Gaussian filter that either attenuated frequencies below ~4.7 cycles per degree (46.875 cycles per image) for high spatial frequency (HSF) images or above ~0.9 cycles per degree (9.375 cycles per image) for low spatial frequency (LSF) images, comparable to filtering used in previous experiments (e.g. Bar et al. [9],; see Figure 1 for examples). The mean (representative of the global luminance) and standard deviation (representative of RMS contrast) of every image was then adjusted to match the mean and standard deviation of the full set of images prior to their filtering in the frequency domain. Thus, each image had an approximately matched global luminance and contrast. A separate set of 76 images were used as practice items.

### Design

There were two factors in the experiment: task (gender classification or living/non-living judgement) and spatial frequency (high or low). There were 296 trials separated into four blocks of 74 trials. Trial order was randomized for each participant. Each object only appeared in one condition per participant; the allocation of objects to conditions was counterbalanced across participants so that, overall, each object appeared in each condition an equal number of times.

Each trial began with one of two cue words presented for 500 ms to indicate which task should be performed on the subsequently presented picture: NATUR (German for “nature”) or NAME (German for “name”). After a period varying randomly between 500 to 800 ms, a picture of an object was then presented for 750 ms. A green fixation cross remained on screen throughout the trial.

Participants were instructed to respond as quickly and as accurately as possible. They used one key for living during the superordinate task and the same key for masculine in the gender-classification task (“Der”). They used another key for non-living or feminine (“die”) items. Participants responded with one hand only, changing response hands halfway through the experiment. Participants were instructed to minimize eye movements and blinking while a fixation cross or the stimulus was on screen.

### EEG recording and analysis

EEG was recorded continuously from 64 scalp locations using active Ag–AgCl electrodes (BioSemi Active-Two amplifier system; Biosemi, Amsterdam, The Netherlands) placed in an elastic cap. This system uses two active electrodes positioned close to the electrode Cz of the international 10–20 system [48] rather than the “ground” electrodes in many other EEG amplifiers: Common Mode Sense (CMS) acts as a recording reference and Driven Right Leg (DRL) serves as ground [49,50]. Four electrooculograms (EOG) – above and below the left eye, and at the outer canthi of each eye - were recorded in order to exclude trials with blinks, large eye movements, and for use in the detection and removal of miniature eye movements. EEG signal was sampled at a rate of 512 Hz and segmented into epochs starting 1 s prior and lasting 1.25 s after picture onset. EEG data processing was performed using the EEGLAB [51] and FieldTrip toolboxes [52] combined with in-house procedures running under the Matlab (The Mathworks, Inc, Natick, Massachusetts) environment. The Fully Automated Statistical Thresholding for EEG Artifact Rejection (FASTER) plug-in for EEGLAB was used for artefact rejection and interpolation of globally and locally artefact contaminated channels [53].

We removed artefacts attributable to miniature eye movements using the method suggested by Keren et al. [45]. In brief, we convolved the radial EOG (the mean of the four EOG channels re-referenced to the posterior parietal electrode Pz; rEOG) with a saccadic-potential filter supplied by Keren et al. [45]. This procedure accentuates the biphasic wave typical of miniature saccades. Subsequently, local peaks greater than three times the root mean square of the rEOG were identified as potential miniature eye movements. Epochs lasting 100 ms before and after the onset of each miniature eye movement were then extracted from the data and submitted to an Independent Component Analysis (ICA). The resulting ICA weights were copied to the full dataset, and components that reflected miniature eye movements or bad channels were rejected. The data were then re-referenced to the average reference before further analysis.

### Behavioural data analysis

Response times (RTs) and errors (%) were analyzed using a repeated-measures ANOVA with the factors of *Task* (Gender – gender classification/basic-level; Superordinate – living/non-living) and *Spatial Frequency* (HSF, LSF). Only reaction times on correct trials were included in this analysis. Responses shorter than 200 ms or longer than 2000 ms were also removed from the analysis. Generalized eta-squared (ƞ^2^_g_.) is reported as a measure of effect size [54–56].

### Evoked and total spectral changes

Time-frequency representations were obtained using sliding-window Fourier transform methods implemented in the FieldTrip toolbox [52]. Prior to further analysis, power line noise was removed using the discrete Fourier transform to estimate and subtract 50 Hz and 100 Hz components from the data. The linear trend and DC component were removed.

High-frequency (30 to 100 Hz in 5 Hz steps) power was estimated using multitapers [57], with a fixed time window of 200 ms and three orthogonal Slepian tapers, yielding a frequency smoothing of ~10 Hz. Evoked gamma band activity (only activity time and phase-locked to stimulus onset; eGBA) was estimated by first averaging the data across trials and then performing time-frequency transformations. Total gamma band activity (both evoked activity and activity neither time- nor phase-locked to stimulus onset; tGBA) was estimated by first performing time-frequency transformations on each trial and then averaging across trials. In both cases, we calculated the percentage change in power relative to a baseline window from 400 ms to 100 ms before stimulus onset.

**Figure 2:**
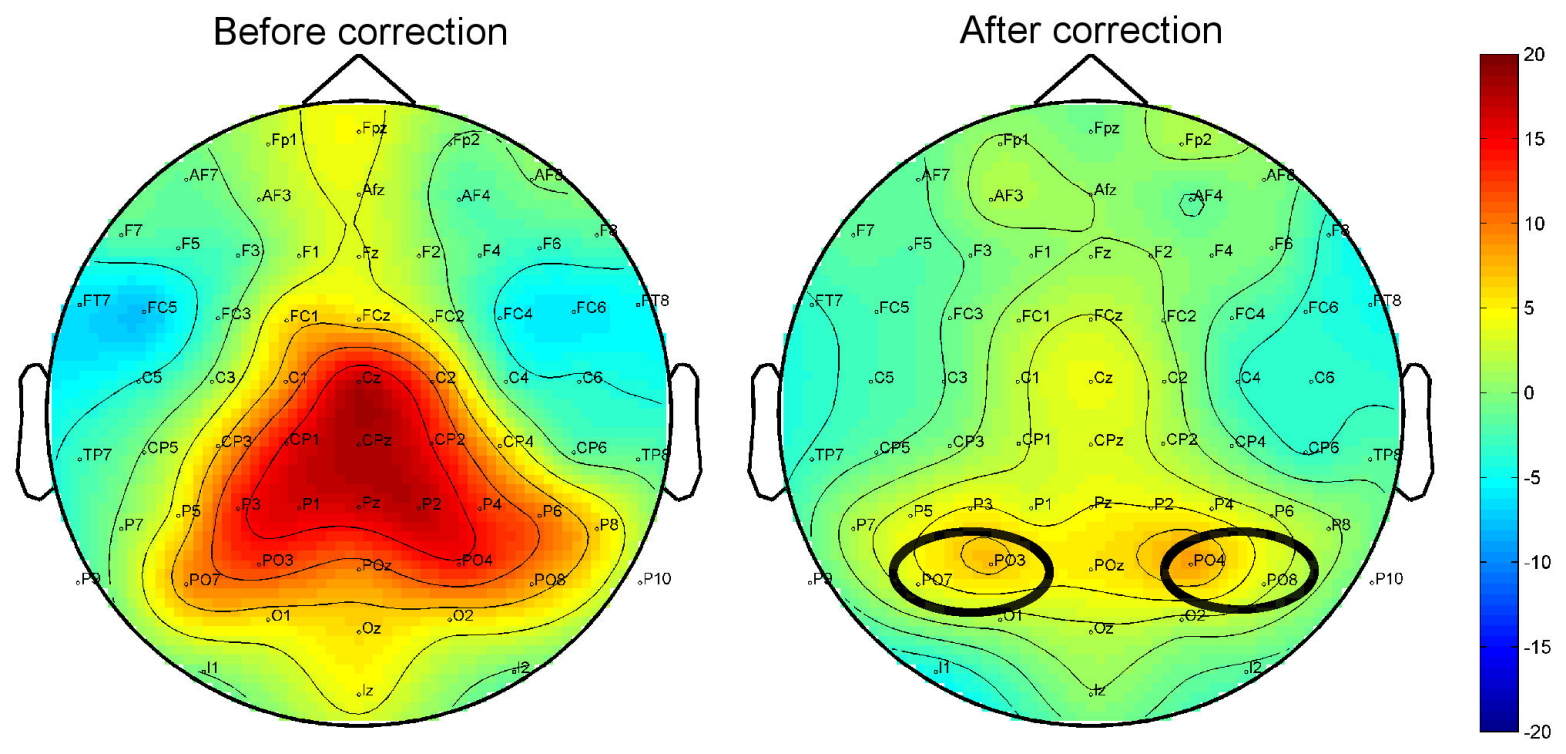
Total gamma band activity topography before and after miniature eye movement correction. Spline interpolated maps depicting percent change in power relative to baseline power of total GBA before and after removal of the miniature eye movement artefact, averaged across all subjects and experimental conditions in the time window between 200 and 700 ms after stimulus onset. Ovals indicate electrodes selected for further analysis of total GBA.

The evoked gamma band response in object recognition paradigms has low variability across individuals, and is typified by a peak in activity between 30–40 Hz around 100 ms after stimulus onset. Thus, we examined average evoked gamma power in the 30-40 Hz range between 50–150 ms. An occipital cluster (electrodes O1, Oz, O2, PO3, POz, and PO4) was selected for eGBA based on previous results [37,38]. Although the induced gamma band response was previously observed as a broad peak between 200 and 300 ms spanning approximately 30-90 Hz, after correction for saccadic spike artefacts it is more commonly seen as a relatively narrow response ongoing from approximately 200 ms after stimulus onset until stimulus offset. The artefact primarily projects to the parietal electrodes [45], and after correction the centro-parietal peak is largely removed (see Figure 2). We thus took the two remaining clusters which showed maximal responses when averaged across all conditions – PO3/4 and PO6/7 – into our analysis. The peak frequency of the induced gamma band response varies across individuals. Thus, we chose peak frequencies for each individual on the basis of plots showing the grand mean response across all conditions for each individual participant, and analysed total gamma power response across the time window from 200 to 700 ms after stimulus onset. Both evoked and total gamma band activity were analysed using repeated measures ANOVA with the factors of *Task* (Gender, Superordinate) and *Spatial Frequency* (HSF, LSF); the analysis of tGBA also included the additional factor *Hemisphere* (Left, Right). ƞ^2^_g_. is reported as a measure of effect size.

### ERP analysis

A 30 Hz low-pass Butterworth filter was applied to the data before ERP analyses. We defined clusters of electrodes and appropriate time windows for ERP analyses on the basis of visual inspection of grand mean plots and using several of the studies reviewed in the introduction for guidance. We used the ERPLAB plug-in for EEGlab to calculate the mean amplitude for each component after subtracting the mean amplitude of a baseline window from 200 ms prior to stimulus onset until stimulus onset from each time point. We assessed the P1 (110-140 ms) and N1 (160-210 ms) at a cluster of central occipital sites (O1, Oz, and O2) associated with early visual processing and at clusters of lateral posterior sites (P7/PO7 and P8/PO8) at which effects of object category are often displayed, typically with enhanced negativity for individual categories such as faces (the N170) [40,58] or cars [59]. We also assessed two later components, the N350 (330-440 ms) and LPC (450-600 ms). Differences between basic-level and superordinate responses have been observed previously in the N350; topographically, it has a left-fronto-central bias [33]. We therefore assessed this component at three clusters of central and fronto-central electrodes at central and lateral locations (FC3/C3, FCz/Cz, and FC4/C4). The LPC is a late component appearing after 400 ms at centro-parietal sites [60] and may reflect secondary categorization processes. We expected these later components to exhibit task but not spatial frequency differences.

The P1, N1, and N350 were analyzed using a repeated-measures ANOVA with the factors of *Task* (Gender, Superordinate), *Spatial Frequency* (HSF, LSF) and *Location* (Left, Central, Right). The LPC was assessed with only the factors Task and Spatial Frequency. Where necessary, Greenhouse-Geisser correction was used in cases of violations of sphericity, and significant effects were examined using post-hoc t-tests with Bonferroni-Holm correction for multiple comparisons. ƞ^2^_g_. is reported as a measure of effect size.

## Results

### Behavioural data

**Figure 3:**
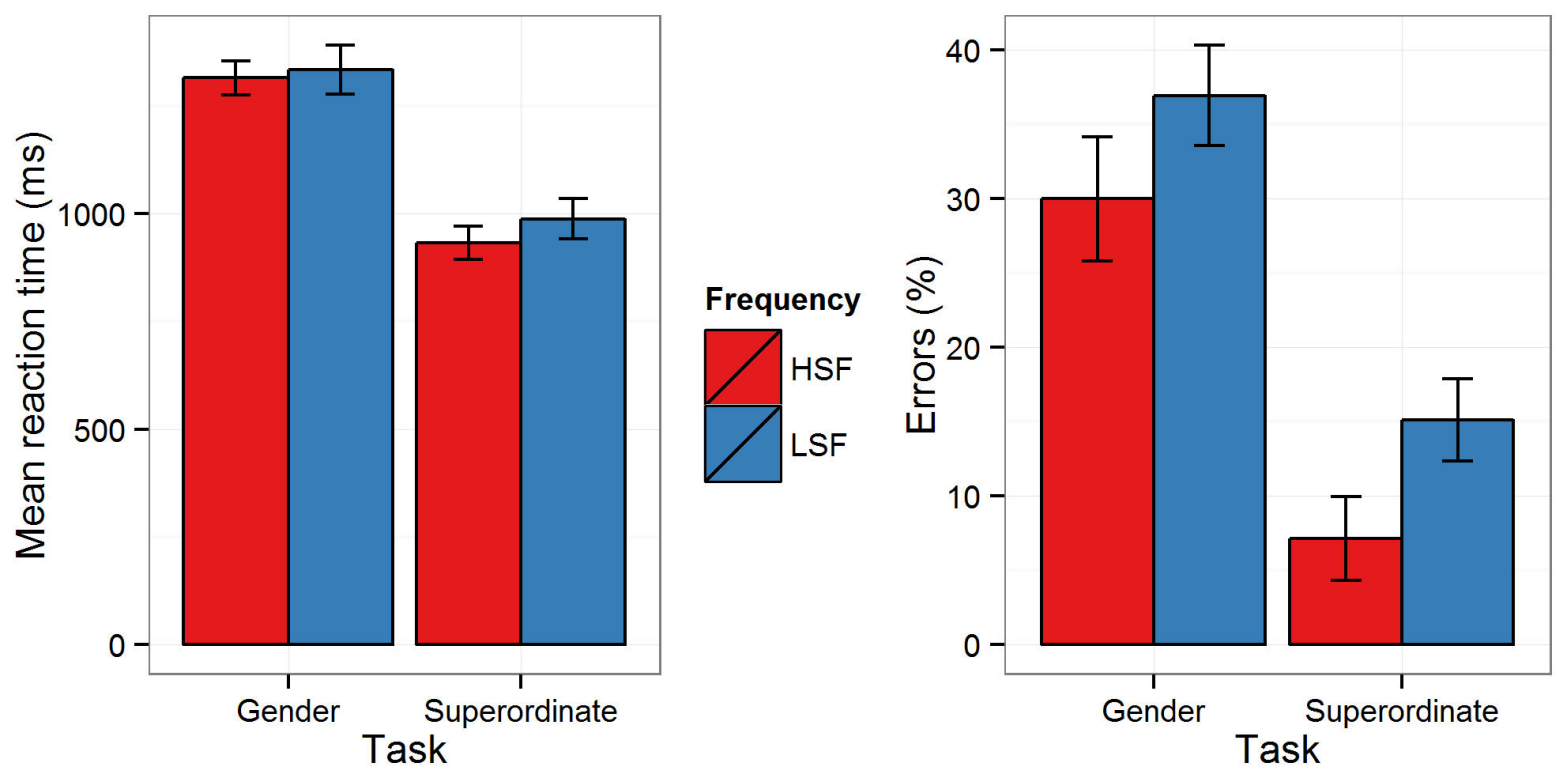
Behavioural data. Mean reaction times (ms; Panel A) and percentage errors (%; Panel B) for the Gender and Superordinate tasks for high spatial frequency (HSF; red bars) and low spatial frequency (LSF; blue bars) images. Error bars show 95% within-participant confidence intervals.

There was a significant main effect of *Task* for both RTs [*F*(1,15) = 138.332, *p* < .001, ƞ^2^_g_. = .3] and errors [*F*(1,15) = 162.497, *p* < .001, ƞ^2^_g_. = .6], with longer RTs and higher errors in the gender classification task than in the living/non-living task. There was also a significant main effect of *Spatial Frequency* for both RTs [*F*(1,15) = 5.213, *p* = 0.04, ƞ^2^_g_. = .004] and errors [*F*(1,15) = 25.858, *p* < .001, ƞ^2^_g_. = .2], with faster responses and fewer errors on HSF than LSF trials. However, the interaction between *Task* and *Spatial Frequency* was not significant for either RTs [*F*(1,15) = 2.164, *p* = .2, ƞ^2^_g_. = .001] or errors [*F*(1,15) = 0.134, *p* = .7, ƞ^2^_g_. < .001] (see Figure 3). Thus, the effect of spatial frequency was comparable in both tasks.

### Evoked gamma-band power

**Figure 4:**
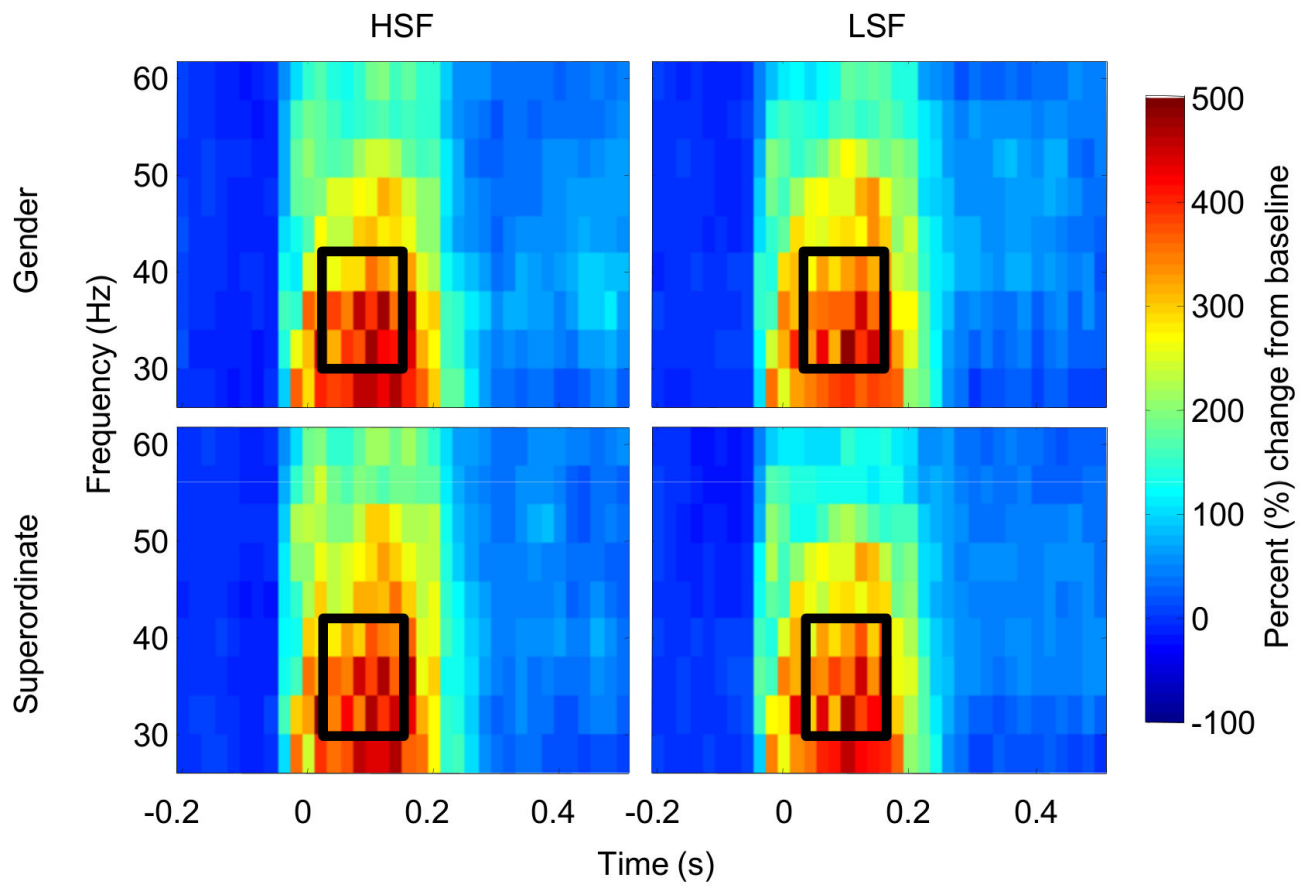
Time-frequency depiction of evoked gamma band activity. Evoked GBA averaged over a cluster of occipital electrodes (see Methods). Left column shows eGBA on HSF trials, right column on LSF trials. Upper row shows eGBA on Gender trials, lower row eGBA on Superordinate trials. Scale is percent change relative to baseline. Black box indicates analysis time and frequency window.

We found no effects of *Task* [*F*(1,15) = .51, *p* = .5, ƞ^2^_g_. = .001], *Spatial Frequency* [*F*(1,15) = .20, *p* = .7, ƞ^2^_g_. < .001], or interactions between them [*F*(1,15) = .002, *p* = .4, ƞ^2^_g_. = .002] in the evoked gamma band range (30-40 Hz) between 50 and 150 ms, see Figure 4.

### Total gamma-band power

**Figure 5:**
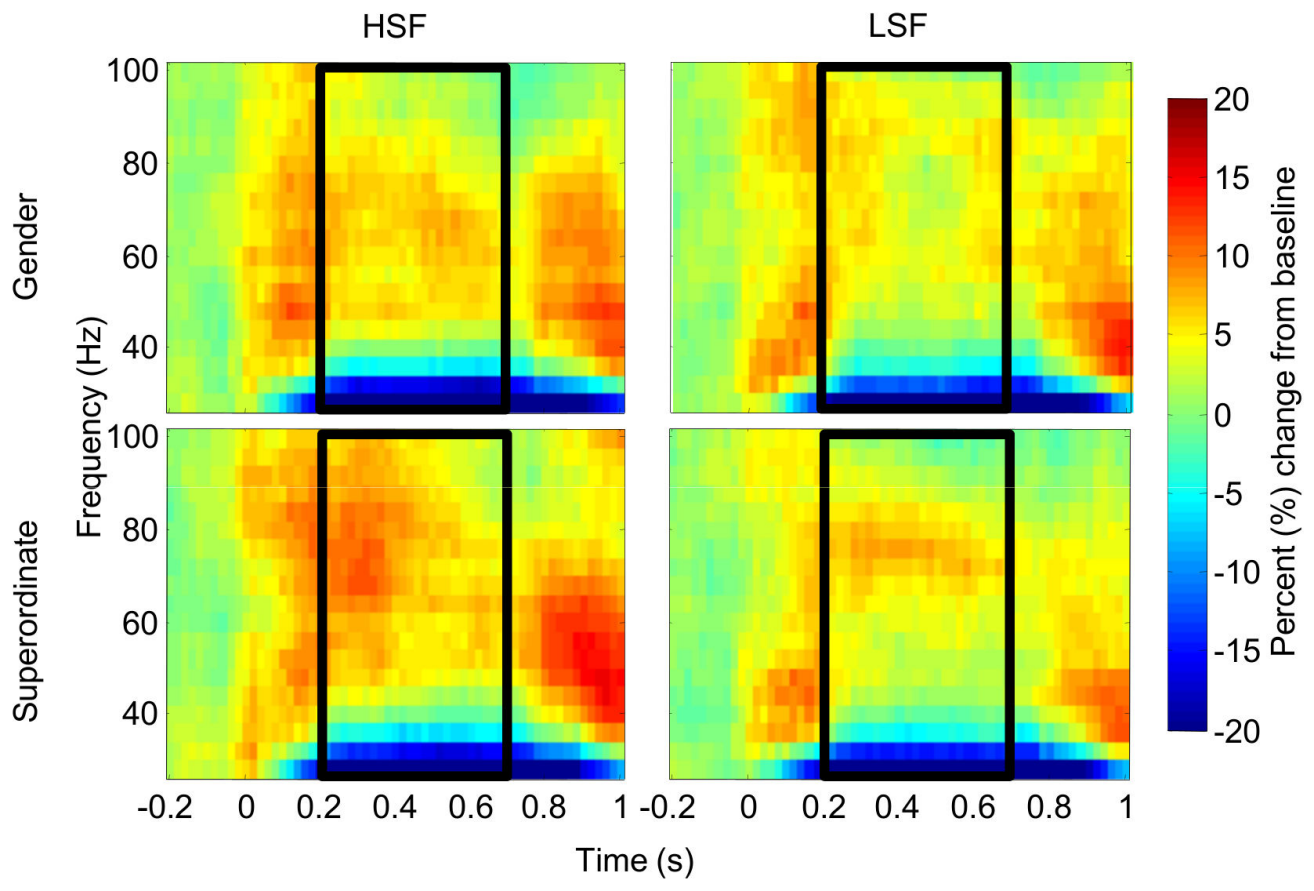
Time-frequency depiction of total gamma band activity. Total high-frequency power averaged across left and right parietal electrodes (see Methods). Left column shows tGBA on HSF trials, right column shows tGBA on LSF trials. Upper row shows tGBA on Gender trials, lower row tGBA on Superordinate trials. Scale is percent change relative to baseline. Black box indicates analysis time window.

**Figure 6:**
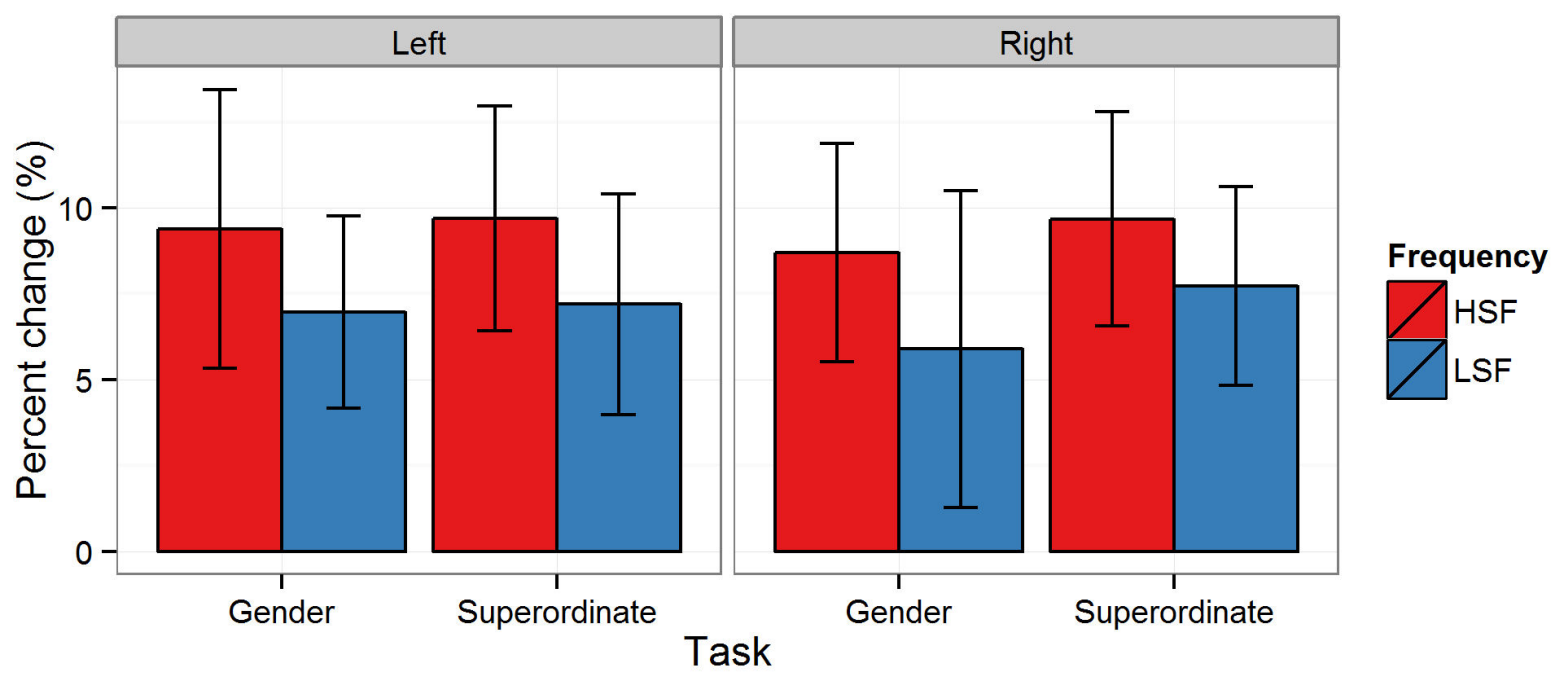
Bar plots showing percent change in tGBA. Percent change from baseline of total gamma band activity from 200–700 ms at left and right occipital clusters for the Gender and Superordinate tasks for HSF (red bars) and LSF (blue bars) images. Error bars indicate 95% within-participant confidence intervals.

Neither the main effect of *Task* [*F*(1,15) = 1.26, *p* = .3, ƞ^2^_g_. = .002], nor the effect of *Spatial Frequency* reached significance [*F*(1,15) = 3.87, *p* = .07, ƞ^2^_g_. = .01], despite a numerical tendency towards greater increases in amplitude for HSF pictures. There was also no main effect of *Hemisphere* [*F*(1,15) = 0.02, *p* = .9, ƞ^2^_g_. < .001]. There were no interactions between any of these factors (all ps > .4]. See Figures 5 and 6.

### ERP analysis

**Figure 7:**
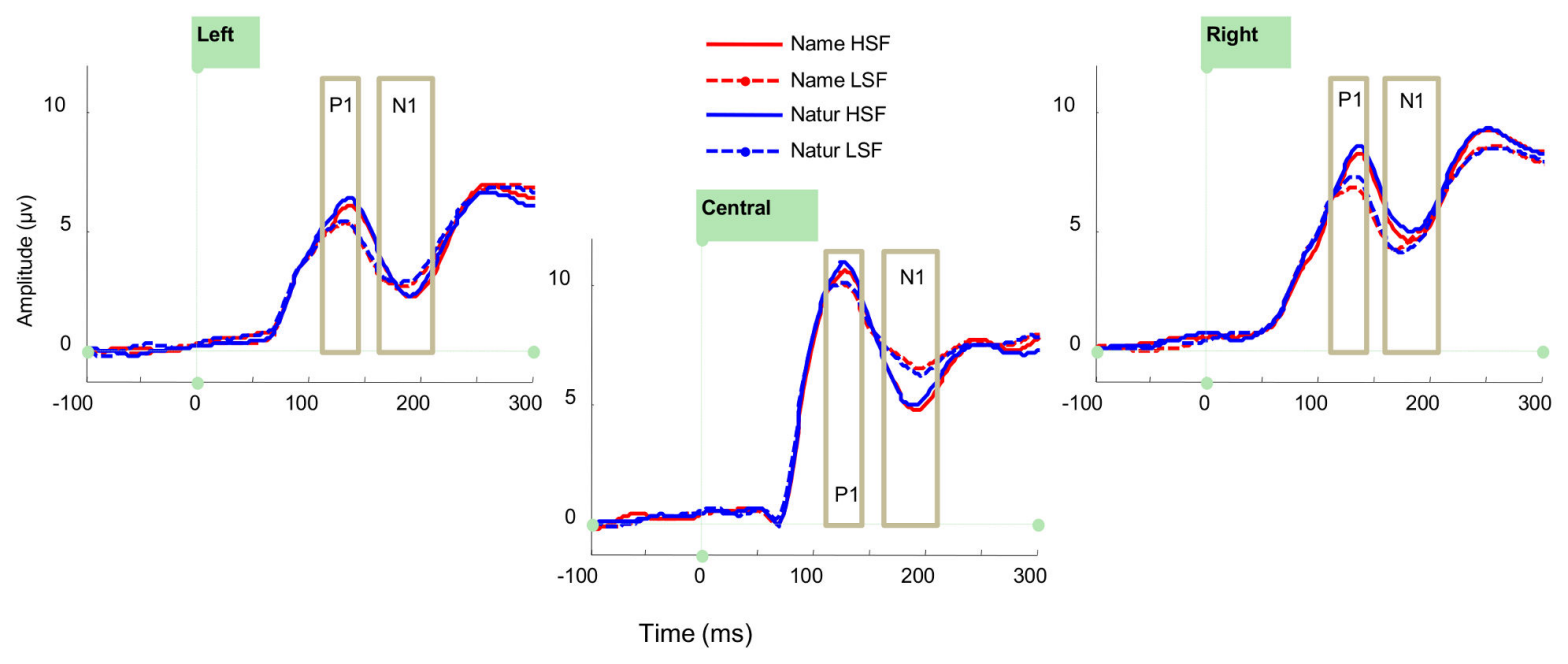
Grand mean ERP plots for the P1 and N1. Grand mean ERPs for P1 and N1 at central occipital and lateral posterior electrode clusters. Boxes indicate time windows selected for further analysis. Red lines show ERPs in the Gender task; blue lines ERPs in the Superordinate task. Solid lines show ERPs to HSF images; dashed lines ERPs to LSF images.

For P1 mean amplitude, there was a main effect of *Location* [*F*(2,30) = 21.68, *p* < .001, ƞ^2^_g_. = .26], with higher amplitudes at the central cluster than at the left or right clusters, and a main effect of *Spatial Frequency* [*F*(1,15) = 5.13, *p* = .04, ƞ^2^_g_. = .008], with higher amplitudes for HSF images. No other main effects or interactions were significant (all ps > .1), see Table 1 and Figure 7.

**Table 1:** Mean amplitudes (± standard error of the mean) of the P1, N1, and N350 ERP components.

		Gender		Superordinate	
Component	Electrode cluster	HSF	LSF	HSF	LSF
P1	Left	5.5±0.6	5.1±0.5	5.8±0.5	5.2±0.5
	Central	10.2±1.1	9.8±0.9	10.5±1.2	9.9±1.0
	Right	7.4±1.0	6.6±0.7	7.6±1.0	7.0±0.7
N1	Left	2.9±0.7	3.1±0.7	3.0±0.8	3.2±0.7
	Central	5.5±1.2	6.9±0.9	5.7±1.3	6.8±0.9
	Right	5.1±1.1	5.0±1.0	5.6±1.2	4.8±1.0
N350	Left	-4.6±0.7	-4.3±0.6	-3.7±0.8	-3.5±0.7
	Central	-6.5±0.8	-5.8±0.8	-5.7±0.9	-5.4±0.8
	Right	-2.7±0.5	-2.6±0.5	-2.7±0.5	-2.8±0.4

Mean amplitudes (± standard error of the mean) of the P1, N1, and N350 ERP components. Note that for the P1 and N1, electrode clusters were at posterior and central occipital sites, whereas for the N350 they were at fronto-central sites.

For N1 mean amplitudes, there was a main effect of *Location* [*F*(2,30) = 8.50, *p* = .001, ƞ^2^_g_. = .1] and an interaction between *Location* and *Spatial Frequency* [*F*(2,30) = 6.81, *p*= .004, ƞ^2^_g_. = .009]. No other effects were significant (all ps > .3). Post-hoc tests on the interaction between Location and Spatial Frequency indicate that there was a marginal effect of spatial frequency (*p* = .06) in the central cluster but not in the right or left clusters (ps = 1). Both HSF and LSF pictures elicited greater negativity in the left cluster than in the central (all *p*s < .01) and the right clusters (all *p*s < .03). The only other significant difference was that LSF pictures elicited more negativity in the right cluster than the central cluster (*p* = .01); see Table 1 and Figure 7.

**Figure 8:**
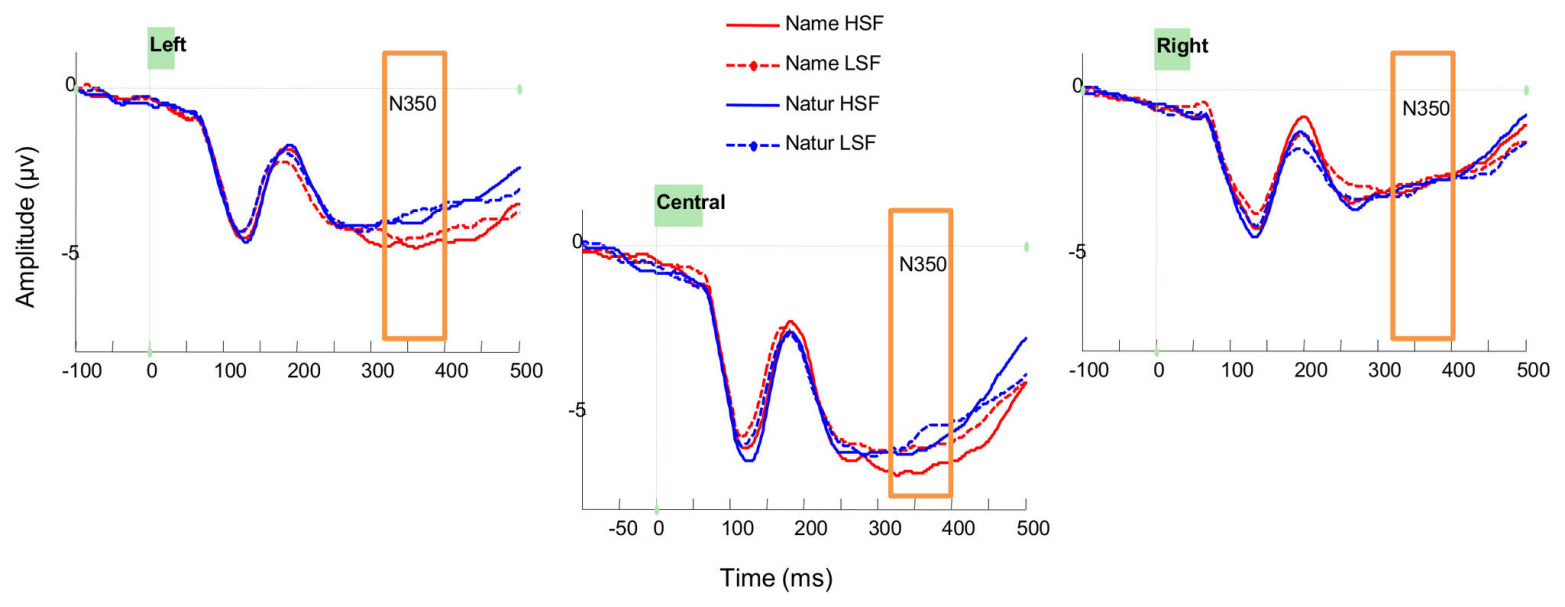
Grand mean ERP plots for the N350. Grand mean ERPs at fronto-central electrodes. The box indicates the time window analysed for the N350. Red lines show ERPs in the Gender task; blue lines ERPs in the Superordinate task. Solid lines show ERPs to HSF images; dashed lines ERPs to LSF images.

For N350 mean amplitudes, there was a main effect of *Location* [*F*(2,30) = 23.55, *p* < .001, ƞ^2^_g_. = .2], with overall lower amplitudes at central sites. There was a significant interaction between *Location* and *Task* [*F*(2,30) = 8.28, *p* = .001, ƞ^2^_g_. = .005]. Post-hoc tests indicated that there was greater negativity in the gender classification task than in the living/non-living superordinate task at left fronto-central sites (*p* = .01), though the same effect was not significant at central (*p* = .06) or at right central sites (*p* = .6); see Table 1 and Figure 8.

The final ERP analysis focussed on the LPC mean amplitude at the parietal electrode Pz. There was a main effect of *Spatial Frequency* [*F*(1,15) = 7.78, *p* = .01, ƞ^2^_g_. = .02], with higher amplitudes for HSF than LSF images. There was also a main effect of *Task* [*F*(1,15) = 10.15, *p* = .006, ƞ^2^_g_. = .01], with lower amplitudes in the gender-classification task than in the superordinate categorization (NATUR) task. The interaction between *Task* and *Spatial Frequency* was not significant (*F*(1,15) = 0.33, p = .5, ƞ^2^_g_. < .001), see Table 2.

**Table 2:** Mean amplitude (± s.e.m.) at electrode Pz for the LPC (450-600ms).

	Mean amplitude (µv)	
Spatial frequency	Gender	Superordinate
HSF	5.0±0.8	5.8±0.9
LSF	4.1±0.8	5.1±0.9

## Discussion

In an EEG experiment, we examined responses to object stimuli containing information at different spatial frequencies during two different tasks: a gender-classification task, which enforces categorization at generally the basic level and has previously been called covert naming [32]; and a living/non-living superordinate categorization task. Previous behavioural evidence suggested that these tasks may rely on different spatial frequency ranges, which may in turn bias information processing towards those ranges. If this is the case (i.e. if spatial frequencies are differently processed depending on task demands) then we would expect early interactions between task and spatial frequency. If, however, both tasks follow the same fixed order of processing (i.e. coarse-to-fine), or depend on similar spatial frequency ranges, then we expected to see no early interactions between spatial frequency and task.

Whereas Collin and McMullen [30] found that the removal of HSF impaired only basic-level categorization, we found that it impaired both basic-level (which was dominant in our gender-classification task) and superordinate object categorization. Participants were slower and less accurate for LSF than HSF stimuli. This is consistent with an account in which both levels of categorization follow the same early processing steps, with one following or contingent upon the other, since the same low-level manipulation influenced performance in both tasks. Our evidence suggests that LSF content alone is less useful for categorizing an object at either the basic or superordinate levels than HSF content alone. Nevertheless, participants were still clearly able to categorize the LSF objects well above chance levels, consistent with an account in which LSF are sufficient to give an initial constraining guess as to an object’s identity [9].

We also found that early ERP components responded somewhat differently to HSF and LSF but did not differ across tasks, and there were no early interactions between task and spatial frequency. In the P1, there were greater positive amplitudes in response to HSF than LSF images. In the N1, only a central occipital cluster showed a hint of a spatial frequency effect, with a greater negativity to HSF than LSF images. Thus, these central sites showed evidence of fewer resources being directed towards analysis of LSF than HSF by this time, as would be expected if processing of LSF were largely complete. Task differences in the ERPs only emerged in the amplitudes of later components (N350; LPC), and although the LPC was also sensitive to spatial frequency, this did not interact with task. Thus, early components reflected perceptual but not cognitive factors, while later components primarily reflected cognitive factors (i.e. categorization task). Evoked GBA was not affected by any of our manipulations, while total gamma band activity showed only marginal but non-significant effects of spatial frequency.

Two previous studies demonstrated a greater N1 negativity for subordinate [33] and entry-level [32] categorization than for basic and superordinate level categorization respectively. We did not replicate such findings with our gender-classification task. Since the N1 is a perceptual component, task-related differences may reflect the availability of task-relevant perceptual attributes. As an example, in Martinovic et al.’s [32] study, stimuli were coloured. Appropriate surface colouring facilitates entry-level categorization [61], and thus may have allowed task differences to emerge. What is most critical here is that task did not interact with spatial frequency, in contrast to Goffaux et al.’s [34] report of modulation of the N170 to faces by a combination of task and spatial frequency. Note that while we may draw an analogy between the specificities of the tasks used here and in Goffaux et al.’s study – both experiments used categorization tasks at a broad level and a more refined level - the tasks were not exact matches. Furthermore, direct comparisons between faces and objects would be necessary to draw strong conclusions regarding differences between face and object processing with respect to task and spatial frequency interactions.

The later effects of task that we observed in the N350 and LPC support their putative role as markers of semantic or secondary categorization processes. We found that the N350, a fronto-central negativity, was more negative for our gender-classification task than for superordinate categorization, whereas Tanaka et al. [33] found greater negativity for their superordinate task than basic-level naming task. Tanaka et al. argued that this component reflected the additional semantic processing required to perform superordinate classification after basic-level categorization. The discrepancy between our results and those of Tanaka et al. may be attributable to task differences. In both tasks participants must access the basic-level name of the object to complete the task. However, in our study, participants indicated the name of an object by reporting its grammatical gender. Drawing on this additional grammatical knowledge may have invoked syntactic processing, which may also exhibit a left fronto-central negativity [62]. Furthermore, Tanaka et al. used a word-picture verification task, in which a word from a given category level was presented before a picture, and participants had to decide if the picture matched the word. It is notable that our task was much harder.

We did not find evidence of task or spatial frequency effects in either evoked or total gamma band activity. Total gamma band activity is thought to reflect the activation of a high-level, abstract object representation, which should be relatively robust to the physical parameters of a given stimulus. Thus, effects of spatial frequency on tGBA should be relatively weak, and we found only a non-significant trend for greater increases in amplitude for HSF than LSF objects. The lack of effects of task on total gamma band activity, which spanned the time window in which effects of task were observed in the N350 and LPC ERP components, suggests that our tasks elicited smaller changes in tGBA than in the ERP measures. Thus, these measures may reflect different underlying processes. Overall, we found little to contradict the account of tGBA reflecting access to a high-level object representation.

In conclusion, our primary hypothesis was that if different spatial frequency information were preferentially processed for different levels of categorization, we would see early interactions between task and spatial frequency. Alternatively, if the initial categorization is made at the same level irrespective of task, then we would expect no interaction in early components, with task differences only emerging in later components. We found no interaction between spatial frequency and categorization task for any of our ERP or time-frequency, or behavioural measures, consistent with models in which initial categorizations are made at the same level of specificity even when the tasks differ. Early ERP P1 and N1 components were affected by spatial frequency but not by level of categorization, with some suggestion that LSF were allocated fewer processing resources by the N1, while later components, the N350 and LPC, were more dependent upon task demands, and may thus provide information regarding predominantly post-perceptual processing of objects. Overall, our results were most compatible with a fixed order of spatial frequency processing and object categorization.
